# Structural Characterization and Rheological and Antioxidant Properties of Novel Polysaccharide from Calcareous Red Seaweed

**DOI:** 10.3390/md20090546

**Published:** 2022-08-25

**Authors:** Faiez Hentati, Latifa Tounsi, Guillaume Pierre, Mohamed Barkallah, Alina Violeta Ursu, Hajer Ben Hlima, Jacques Desbrières, Didier Le Cerf, Imen Fendri, Philippe Michaud, Slim Abdelkafi

**Affiliations:** 1 INRAE, URAFPA, Université de Lorraine, F-54000 Nancy, France; 2 Clermont Auvergne INP, CNRS, Institut Pascal, Université Clermont Auvergne, F-63000 Clermont-Ferrand, France; 3 Laboratoire de Génie Enzymatique et Microbiologie, Équipe de Biotechnologie des Algues, Département Génie Biologique, Ecole Nationale d’Ingénieurs de Sfax, Université de Sfax, Sfax 3038, Tunisia; 4 IPREM, Helioparc Pau Pyrénées, Université de Pau et des Pays de l’Adour, 2 Avenue P. Angot, CEDEX 9, 64053 Pau, France; 5 UNIROUEN, INSA Rouen, CNRS, PBS, Normandie University, 76000 Rouen, France; 6 Laboratoire de Biotechnologie des Plantes Appliquée à l’Amélioration des Cultures, Faculté des Sciences de Sfax, Université de Sfax, Sfax 3038, Tunisia

**Keywords:** sulfated xylogalactan, *Jania adhaerens*, structure, flow behavior, viscoelasticity, antioxidant activity

## Abstract

A novel sulfated xylogalactan (JASX) was extracted and purified from the rhodophyceae *Jania adhaerens*. JASX was characterized by chromatography (GC/MS-EI and SEC/MALLS) and spectroscopy (ATR-FTIR and ^1^H/^13^C NMR) techniques. Results showed that JASX was constituted by repeating units of (→3)-β-d-Gal*p*-(1,4)-3,6-α-l-AnGal*p*-(1→)_n_ and (→3)-β-d-Gal*p*-(1,4)-α-l-Gal*p*-(1→)_n_ substituted on *O*-2 and *O*-3 of the α-(1,4)-l-Gal*p* units by methoxy and/or sulfate groups but also on *O*-6 of the β-(1,3)-d-Gal*p* mainly by β-xylosyl side chains and less by methoxy and/or sulfate groups. The M_w_, M_n_, Đ, [*η*] and C* of JASX were respectively 600 and 160 kDa, 3.7, 102 mL.g^−1^ and 7.0 g.L^−1^. JASX exhibited pseudoplastic behavior influenced by temperature and monovalent salts and highly correlated to the power-law model and the Arrhenius relationship. JASX presented thixotropic characteristics, a gel-like viscoelastic behavior and a great viscoelasticity character. JASX showed important antioxidant activities, outlining its potential as a natural additive to produce functional foods.

## 1. Introduction

Marine red seaweeds (Rhodophyta) are rich sources of bioactive compounds, especially sulfated galactans (carrageenans, agarans, and agaroids) having diverse biological activities and industrial applications [1,2]. These polysaccharides are constituted of alternating (→3)-β-Gal*p* (A unit) and (→4)-α-Gal*p* (B unit) (and/or 3,6-α-AnGal*p*) residues and can be substituted by sulfate, *O*-glycosyl, pyruvic acid ketals, and methoxy groups, side chains and terminal residues.

Carrageenans are the food industry’s third-largest hydrocolloid (after gelatin and starch) and consist of repeating 3-β-d-Gal*p* and 4-α-d-Gal*p* (or 3,6-α-d-AnGal*p*) units. Agarans are sulfated galactans having B residues of the l-series ((1,4)-α-l-Gal*p*). These are classified into two groups, i.e., agaroids (weak gelling agents) and agars (high gelling agents) [1], depending on the content of 3,6-α-d-AnGal*p* residues and sulfate groups. Agaroids are weakly gelling polymers, which can be divided into (i) funorans and (ii) porphyrans. Funorans are constituted of alternating A (6-*O*-SO_3_^−^) and B (2-*O*-SO_3_^−^) units and are widely used in the adhesives field. These structural diversities largely affect the physicochemical (macromolecular conformation) and rheological properties (flow and dynamic characteristics) of these polymers [3,4]. 

Sulfated galactans and/or xylogalactan isolated from red seaweeds exhibit several biological properties, including anticancer, anticoagulant, antiproliferative, antithrombotic, antioxidant, gastroprotective, anti-inflammatory, immunomodulatory and antinociceptive ones [5,6]. Sulfated polysaccharides are currently used in food (food texturizers), and in the cosmetic, nutraceutical, and pharmaceutical fields as thickening, gelling, and stabilizing agents. [1,5]. Bilan and Usov [7] illustrated that sulfated galactans and/or xylogalactan produced by red seaweeds of Corallinales order was less studied, most likely due to their thick calcareous cover, which reduce polysaccharide extraction yields. Different polysaccharide structures obtained from species of the Corallinales order (e.g., *Jania* rubens, *Corallina officinalis*, *Corallina pilulifera*, *Bossiella orbigniana*, *Joculator maximus*, *Bossiella cretacea*, and *Lithothamnion heterocladum*) have been reported [8,9,10,11]. They have an agaran structure constituted of (→3)-β-d-Gal*p*-(1→4)-α-l-Gal*p*-(1→)_n_ and substituted by β-d-Xyl*p* side chains, methoxy and/or SO_4_^2−^ groups with the presence of (1→(3,6)-α-l-AnGal*p* units [7,8,9,12].

*Jania adhaerens*, member of Corallinales order and Corallinaceae algal family, is widely distributed along the Atlantic and Mediterranean coasts, mostly mixed with the brown seaweed *Cystoseira crinita.* [13,14]. For the first time, our study evaluated the structural characterization (using chromatographic and spectroscopic methods), the physicochemical, the rheological investigations as well as the antioxidant activities of a novel sulfated xylogalactan (JASX) to investigate its potential as a hydrocolloid.

## 2. Results and Discussion

### 2.1. Structural Analysis of JASX

#### 2.1.1. Extraction Yield and Biochemical Composition

Table 1 shows the extraction yield as well as the global biochemical composition of JASX. This later exhibited an extraction yield of 5.25% (*w*/*w*), which is close to those obtained for the extraction of polysaccharides from the red seaweeds *Gracilaria birdiae* (6.50%) and *Sarconema filiforme* (6.0%) [15,16,17]. 

From Table 1, JASX consists of carbohydrates (73.52%), neutral sugar (68.02%), 3,6-AnGal residues (19.53%) with minor amounts of phenolic compounds (0.66%) and protein (0.64%). These findings were consistent with previous studies on sulfated galactans isolated from red seaweed species *J. rubens*, *C. officinalis*, and *H. durvillei* [1,10,18]. 

#### 2.1.2. Monosaccharide Analysis

The GC/MS analysis (after acidic hydrolysis and derivatization) demonstrated that JASX was principally constituted of galactose (Gal, 73.06%), xylose (Xyl, 16.66%), glucose (Glc, 8.46%) and small amounts of glucuronic acid (GlcA, 1.81%). This result suggested that JASX was a highly sulfated xylogalactan as described in the literature for sulfated galactans/xylogalactans of the Corallinales order such as *J. rubens* and *C. officinalis* [10,12]. JASX’s main constitutive monosaccharides were Xyl*p* and Gal*p*, with a Gal*p*/Xyl*p* ratio of 4.39. (Table 2). 

These results were consistent with studies performed on xylogalactans isolated from *J. rubens* (2.6–3.5), *C. officinalis* (2.9–4.4), and *B. orbigniana* (2.9–4.4). This value of Galp/Xylp ratio appeared higher than those found in xylogalactans isolated from *L. heterocladum* (1.5–3) [11], but was lower than those found in sulfated galactans isolated from *Spyridia hypnoides* and *H. durvillei*, which had high Gal*p*/Xyl*p* ratios (>10) [1,19].

#### 2.1.3. ATR-FTIR Spectroscopy

The FTIR spectra of JASX (sulfated fraction) and JADX (desulfated fraction) were obtained (Supplementary Figure S1A,B). Bands at around 3344 and 2926 cm^−1^ were assigned respectively to OH- stretching and CH- asymmetric vibrations of JASX. The weak absorption bands at 1640 cm^−1^ (carboxylate groups -COO-) and at 1422 cm^−1^ (ester carbonyl groups C=O) of acid residues confirmed JASX’s weak electrolytic character (6.55%). The characteristic bands at around 1240, 1148, 1029, 900 and 880 cm^−1^ were ascribed to the FTIR footprints of agar-like polysaccharide. The asymmetric O=S=O stretching vibration of the sulfate ester [20] was observed at the 1240 cm^−1^ peak. The signal at 1148 cm^−1^ was attributed to C-O glycosidic band vibrations, whereas the strong absorption band at 1029 cm^−1^ was attributed to the carbohydrate stretching vibrations (pyranose ring of Gal*p* residues). The weak signal near 900 cm^−1^ was attributed to the vibration C−O−C bridge in 3,6-α-l-AnGal*p* units [21]. Peaks at 900–570 cm^−1^ were expanded to determine the sulfate group position in JASX (agaran). Signals at 900 and 880 cm^−1^ (agar specific bands) were assigned respectively to 3-*O*-SO_3_^−^ and/or 2-*O*-SO_3_^−^ groups present in α-l-Gal*p* units [22], whereas bands at 925 and 820 cm^−1^ were attributed to (6-*O*-SO_3_^−^) stretching of sulfate groups on β-d-Gal*p* residues and to the 3,6-AnGal*p* (2-*O*-SO_3_^−^) units, respectively [5]. JADX showed a drop in intensities for the characteristic signals (region 500–1250 cm^−1^) corresponding to sulfate groups after desulfation (see Section 3.4.). Colorimetric assays (around 2%) confirmed the desulfation yield of 54.60% (*w*/*w*).

#### 2.1.4. NMR Spectroscopy

**Figure 1:**
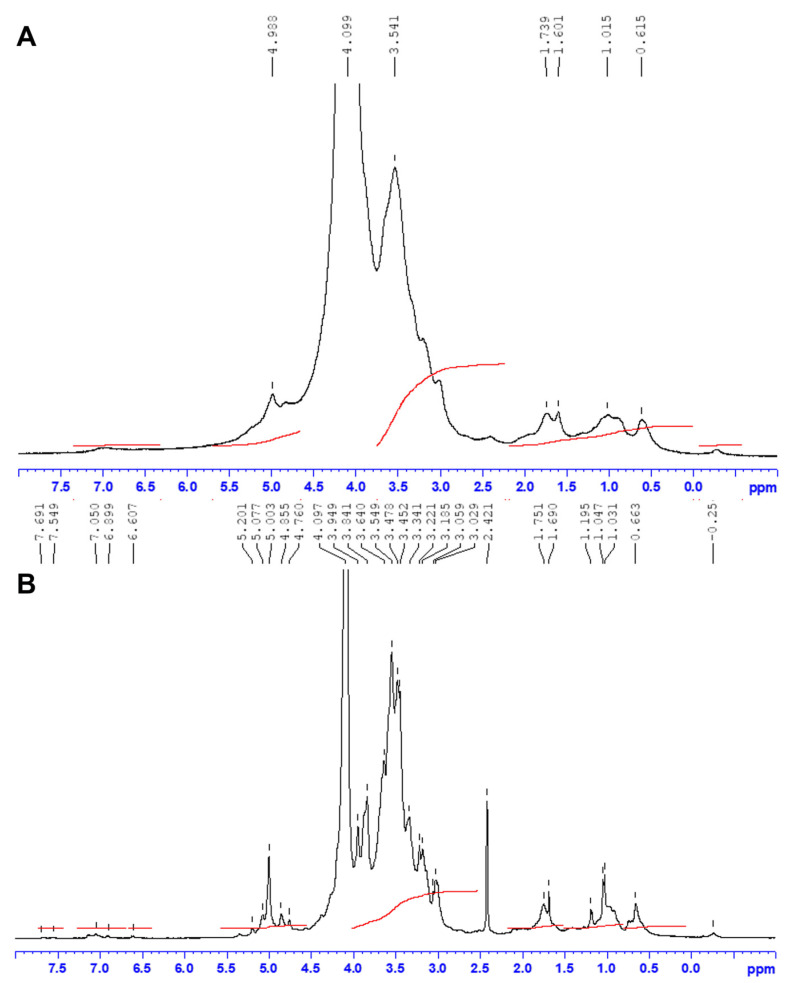
^1^H-NMR spectra of JASX (**A**) and JADX (**B**) fractions. The analyses were recorded for polysaccharides in D_2_O (40 g.L^−1^) at 60 °C.

The ^1^H-NMR and ^13^C-NMR analyzes were also performed to determine the precise structure and sulfation pattern of JASX. Several assignments were deduced by comparing the NMR spectra of JASX and JADX (Figure 1A,B) with the ^1^H/^13^C NMR results of (i) a sulfated galactan isolated from *J. maximus* having a main chain of →3)-β-d-Gal*p*-(1,4)-α-l-Gal*p*-(1→ substituted on *O*-2 and *O*-3 of α-(1,4)-l-Gal*p* and on *O*-6 of β-(1,3)-d-Gal*p* by short side chains and β-d-Xyl*p* terminal units [9]; (ii) a sulfated xylogalactan-rich fraction isolated from *J. adhaerens* (alkaline extraction) constituted by repeating units of →3)-β-d-Gal*p*-(1,4)-α-l-Gal*p*-(1→)_n_ substituted on *O*-6 of (1,3)-β-d-Gal*p* as well as on *O*-2 and *O*-3 of (1,4)-α-l-Gal*p* units by β-xylosyl side chains, 3,6-α-l-AnGal*p* units*,* sulfate and methoxy groups [14]; (iii) a *J. rubens* sulfated xylogalactan showing main backbone of [→4)-α-l-Gal*p*-(1,3)-β-d-Gal*p*-(1→] branched on *O*-6 of the β-d-Gal*p* unit and on *O*-2 and/or *O*-3 of the α-l-Gal*p* (or 3,6-α-l-AnGal*p*) units by β-d-Xyl*p* side stubs, sulfate and methoxy groups, respectively [12]; (iv) a *C. officinalis*-derived sulfated xylogalactan with an alternating [→4)-l-Gal*p*-(1,3)-β-d-Gal*p*-(1→] backbone lacking 3,6-AnGalp, with β-d-Gal*p* units completely substituted on *O*-6, primarily by β-d-Xyl*p* and sulfate groups, whereas the α-l-Gal*p* was partially substituted on *O*-2 and *O*-3 by sulfate and methoxy groups [8]; (v) a *C. pilulifera* sulfated xylogalactan composed of [→3)-β-d-Gal*p*-(1,4)-α-l-Gal*p*-(1→] alternating units substituted by only β-d-Xyl*p* units in *O*-6 of β-d-Gal*p* units, while methoxy and sulfate groups occupy *O*-6 of β-d-Gal*p* and *O*-2 and *O*-3 of α-l-Gal*p* units [23]; (vi) a sulfated xylogalactan extracted from *Gracilaria caudata* constituted of [→3)-β-d-Gal*p* and →4)-3,6-α-l-AnGal*p* alternating units highly substituted by methoxy groups and pyruvic acid acetal; (vii) a complex xylogalactan from the red seaweed *L. heterocladum* with an alternating units of →3)-β-d-Gal*p* and →4-α-l-Gal*p* highly branched principally on *O*-6 position of the β-d-Gal*p* units by β-d-xylosyl side stubs, and less with sulfate or/and methoxy groups, and on *O*-2 position by methoxy and/or sulfate groups of the α-l-Gal*p* and/or 3,6-α-l-AnGal*p* residues [11], and finally, (viii) a *B. cretacea* xylogalactan presenting a similar main backbone consisting of [→3)-β-d-Gal*p*-(1,4)-α-l-Gal*p*-(1→] partially sulfated on *O*-6 position of β-(1,3)-d-Gal*p* residues, a characteristic unique for this algal species [24].

**Figure 2:**
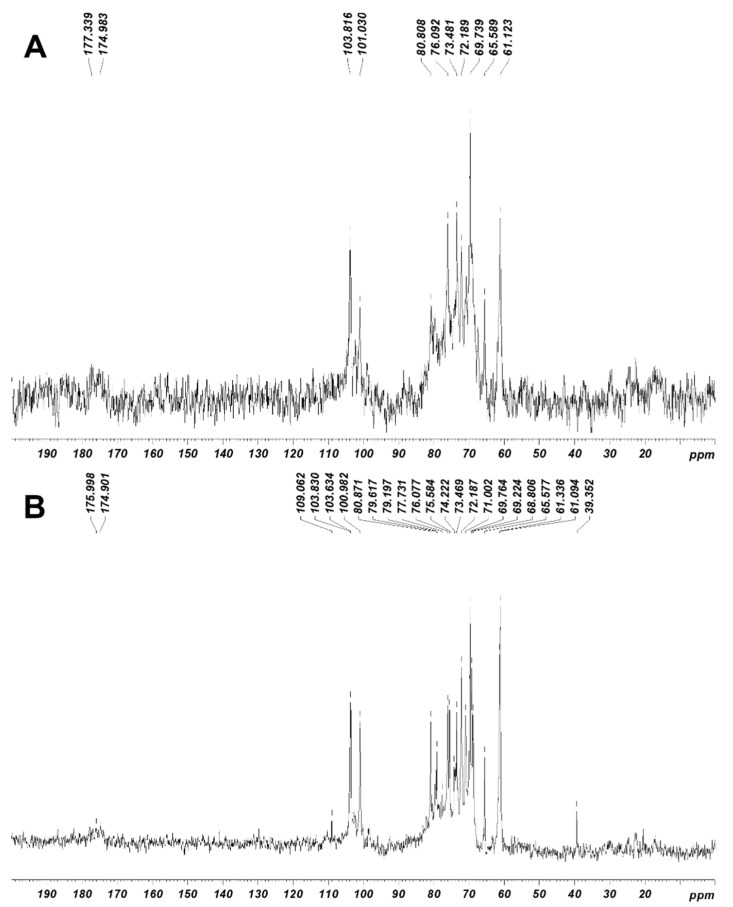
^13^C-NMR spectra of JASX (**A**) and JADX (**B**) fractions. The analyses were recorded for polysaccharides in D_2_O (40 g.L^−1^) at 60 °C.

The ^1^H–NMR (Figure 1A,B) and ^13^C–NMR (Figure 2A,B) assignments of JASX and JADX spectra revealed a great multiplicity degree, suggesting a wide diversity in the β-d-Gal*p* and α-l-Gal*p* main chain, which appeared to be similar to those previously published for other red algae of the Corallinales order such as *C. officinalis*, *B. cretacea*, *J. maximus*, *J. rubens* and *L. heterocladum* [8,11,12,24]. 

Signals related to anomeric regions are located in the range δ 100–115 ppm for ^13^C-NMR and δ 4.1–5.5 ppm for ^1^H-NMR for both JASX and JADX fractions. The signals at around δ 102/4.85 in ^13^C- and ^1^H-NMR, respectively, were ascribed to anomeric carbon and proton of →3)-β-d-Gal*p*-(1,4)-3,6-α-l-AnGal*p*-(1→ residues, while, the signals at δ 103.81/4.76 were attributed to the →3)-β-d-Gal*p*-(1,4)-α-l-Gal*p*-(1→ substituted units, confirming the main chain structure of JASX [12,25]. The signals at δ 101.03/4.98 ppm were attributed to anomeric proton of α-(1,4)-l-Gal*p* units substituted at *O*-3 position mainly by SO_4_^2−^ groups and rarely with methoxy groups [25]. The resonance at δ 101.09/4.98 ppm was already attributed to the 3-*O*-methyl-α-l-Gal*p* unit [12], but in this case, a SO_4_^2−^ group could be present at this position compared to the JADX spectrum. A weak signal at around 98.50 ppm in the ^13^C–NMR spectrum (Figure 2B) could be assigned to the α-Glc*p* residues, which was in accordance with the monosaccharide composition (8.46% *w*/*w*).

**Figure 3:**
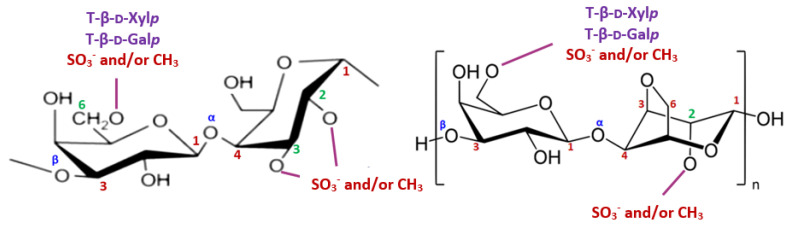
The proposed structure for JASX.

The resonances at around δ 98.2/5.07 and 99.8/5.00 ppm were mainly affected by 3,6-An-α-l-Gal*p* (2-*O*-SO_3_^−^ or 2-*O*-methyl ether), while the assignments at δ 100.98/5.20 ppm were attributed to the H–1 of 2-*O*-methyl-α-l-Gal*p* residues [10,12]. The 3-Me-α-l-Gal*p* and α-l-Gal*p* residues were identified respectively at δ 103.63 ppm (^13^C-NMR) and δ 4.80 ppm (^1^H–NMR), while the signal at around δ 101.2 ppm was ascribed to the α-(1,4)-linked l-Gal*p* (2-*O*-SO_3_^-^) units [10,12]. The ^13^C– and ^1^H–NMR chemical shifts corresponding to β-d-xylosyl units (H1–H5′ and C1–C5) were observed in the range of 3.30–4.30 ppm for ^1^H-NMR and 61–85 ppm for ^13^C–NMR [10,12]. We conclude that JASX presented an original xylogalactan structure (Figure 3) compared to the literature for other sulfated polysaccharides produced by Corallinales.

#### 2.1.5. Molar Mass and Macromolecular Characteristics of JASX

From the SEC-MALLS-Viscosity analysis (Table 2), JASX was characterized by a mass-average molecular mass (M_w_) and a number-average molecular mass (M_n_) of 600 × 10^3^ and 160 × 10^3^ g/mol, respectively. High M_w_ values were similar with previous research on sulfated polysaccharides obtained from some rhodophyceae, having values greater than 1 × 10^5^ g/mol [5,26,27]. These findings remained lower than those reported for xylogalactans isolated from Corallinales order, such as *J. rubens*, *B. orbigniana* and *C. officinalis* [10,12]. The polydispersity index (Đ = M_w_/M_n_) value of 3.7 could be due to the presence of a weak short chain in the JASX structure, which decreased the M_n_ value and therefore slightly increased the polydispersity [14]. According to Khan et al. [28], polymer intrinsic viscosity [*η*] (mL.g^−1^), indicating the capacity of polymers to enhance the viscosity of fluids, depends on several physicochemical characteristics such as its molecular conformation, type and degree of ramifications, molar mass, and solvent properties. JASX exhibited [*η*] and hydrodynamic radius R_h_ values of 102 mL.g^−1^ and 17.2 nm, suggesting a flexible random coil conformation with a value of Mark–Houwink–Sakurada ([*η*] *=* K.M_w_^α^) exponent α ranging between 0.5 and 0.8 [4].

### 2.2. Rheological Properties of JASX

#### 2.2.1. Steady-Shear Flow Measurements of JASX

**Figure 4:**
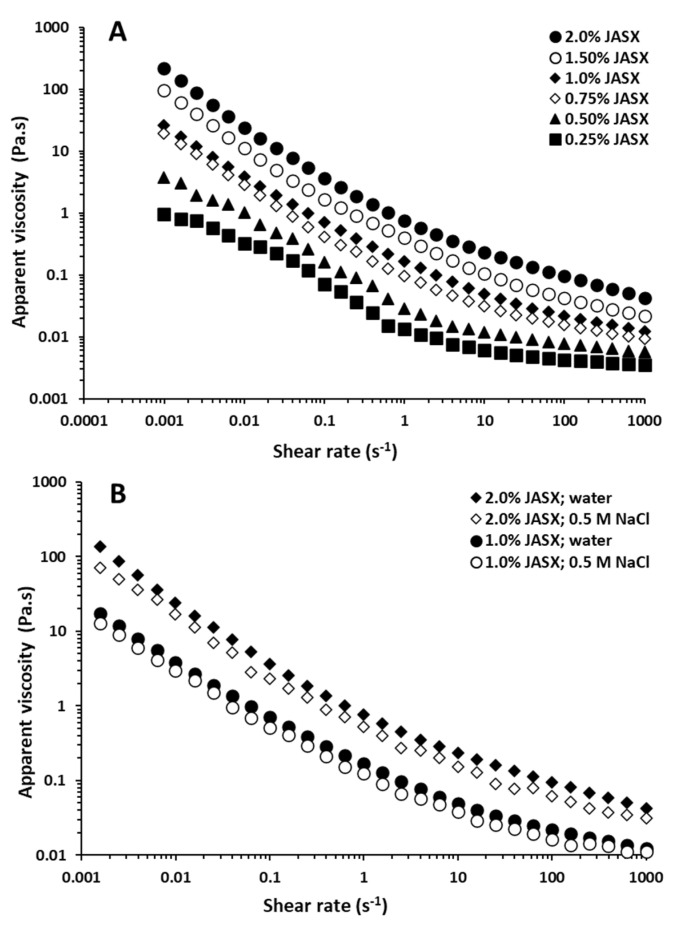
(**A**) Flow behavior of aqueous JASX solutions at different concentrations ranging from 0.25 to 2.0% (*w*/*v*) at 25 °C. (**B**) Influence of salts (0.5 M NaCl) on the apparent viscosity of JASX solutions ranging from 1.0 to 2.0% (*w*/*v*) at 25 °C.

The flow curves (apparent viscosity (*η*) vs. shear rate ($\dot{\gamma }$)) of aqueous JASX solutions (0.25–2.0%, *w*/*v*) at 25 °C are shown in Figure 4A. JASX solutions in water presented non-Newtonian shear-thinning (pseudoplastic) behavior since *η* (Pa.s) decreased with increasing $\dot{\gamma }$ (s^−1^) from 0.001 to 1000 s^−1^, suggesting that intermolecular entanglements of JASX in water tended to rise with increasing polymer concentration. This observation was consistent with previous findings obtained for other sulfated galactans and/or xylogalactans isolated from *H. durvillei* and *G. birdiae* and [1,22].

The Ostwald-de Waele (power-law) model was achieved to fit the rheological data of JASX (0.25–2.0%, *w*/*v*) at 25 °C. Results showed that JASX presented values of flow behavior index *n* lower than 1 (*n* < 1), confirming the pseudoplastic fluid property (Table 3). The rise in polymer concentration decreased the *n* values and increased the coefficient of consistency (*k*). The coefficients of determination (*R*^2^) values superior to 0.99 showed that JASX (0.25–2.0%, *w*/*v*) flow behavior in water was well described by the Ostwald-de Waele model [29].

**Figure 5:**
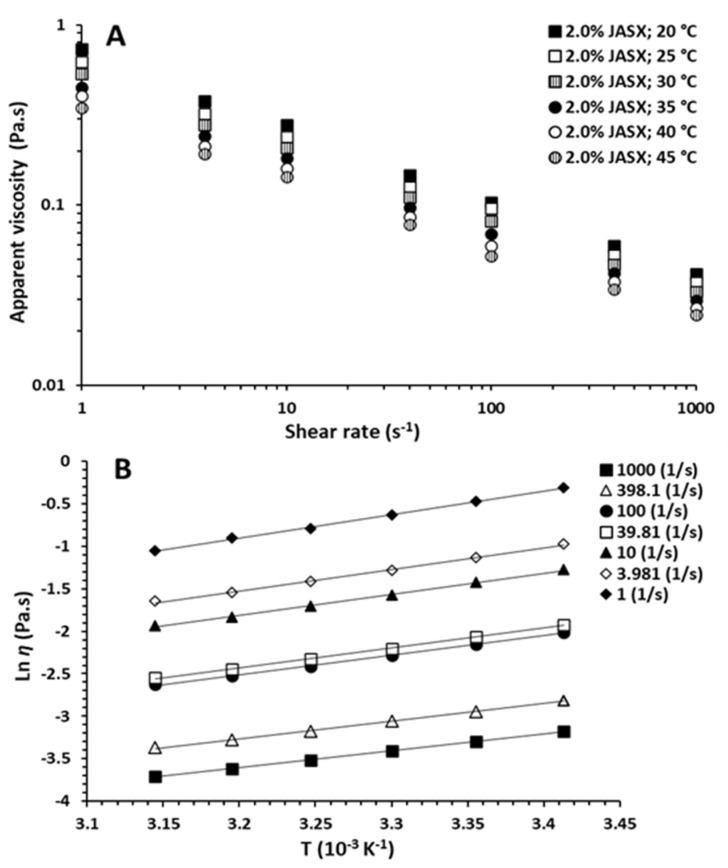
(**A**) Steady-shear flow curves for aqueous JASX solutions of 2.0% (*w*/*v*) at different temperatures ranging from 20 to 45 °C. (**B**) Dependence of viscosity on temperatures (20–45 °C) for 2.0% (*w*/*v*) JASX solutions at shear rates ranging from 1 to 1000 s^−1^. Solid lines representing the fitted curves based on the Arrhenius–Frenkel–Eyring relationship.

The effects of adding monovalent salts (0.5 M NaCl) and increasing temperature (from 20 to 45 °C) on flow properties of JASX (1.0–2.0%, *w*/*v*) are presented in Figure 4B and Figure 5A. From Figure 4B, JASX exhibited pseudoplastic properties, and the *η* decreased with adding 0.5 M NaCl solutions. According to Hentati et al. [29], the intermolecular electrostatic repulsions number and/or the junction zones complexity between JASX molecules decreased with adding monovalent cations, demonstrating that this polysaccharide adopted a more compact conformation due to its low uronic acid content and the lack of sulfate groups in its complex and highly branched backbone. The data shown in Table 3 confirmed that the Na^+^ ions slightly decreased the pseudoplastic character of JASX solutions by decreasing *k* values and increasing *n* values.

Regarding temperature effect, the *η* decreased with the rise in temperature from 20 to 45 °C, and a non-Newtonian shear-thinning behavior (*n* < 1) was observed (Figure 4A). 

These results could be linked to the increase in intermolecular distances by minimizing entanglements and interactions between JASX chains (thermal expansion phenomenon) [4]. The Arrhenius–Frenkel–Eyring equation (Section 3.6.2) was applied to verify the temperature dependency, which could be attributed to the conformational modification of the JASX backbone or to a less stable molecular equilibrium transition accompanied by the thermal expansion phenomenon. 

As illustrated in Figure 5B, the high values of *E_a_* implied that JASX at 2.0% (*w*/*v*) was very sensitive to the temperature. Table 4 showed that the flow activation energy (*E_a_*) values were dropped with the rising $\dot{\gamma }$, and the *η* was decreased by rising temperature, suggesting then that the JASX in water flowed more readily under shearing [30]. These observations demonstrated that the rise in temperature caused (i) an increase in the energy dissipation motions of macromolecules, (ii) a breakdown of the weak energy bonds, and (iii) then a drop in the *E_a_* values, showing that these results were a kind of non-Newtonian pseudoplastic fluid property for JASX [29].

#### 2.2.2. Dynamic Viscoelastic Properties of JASX

**Figure 6:**
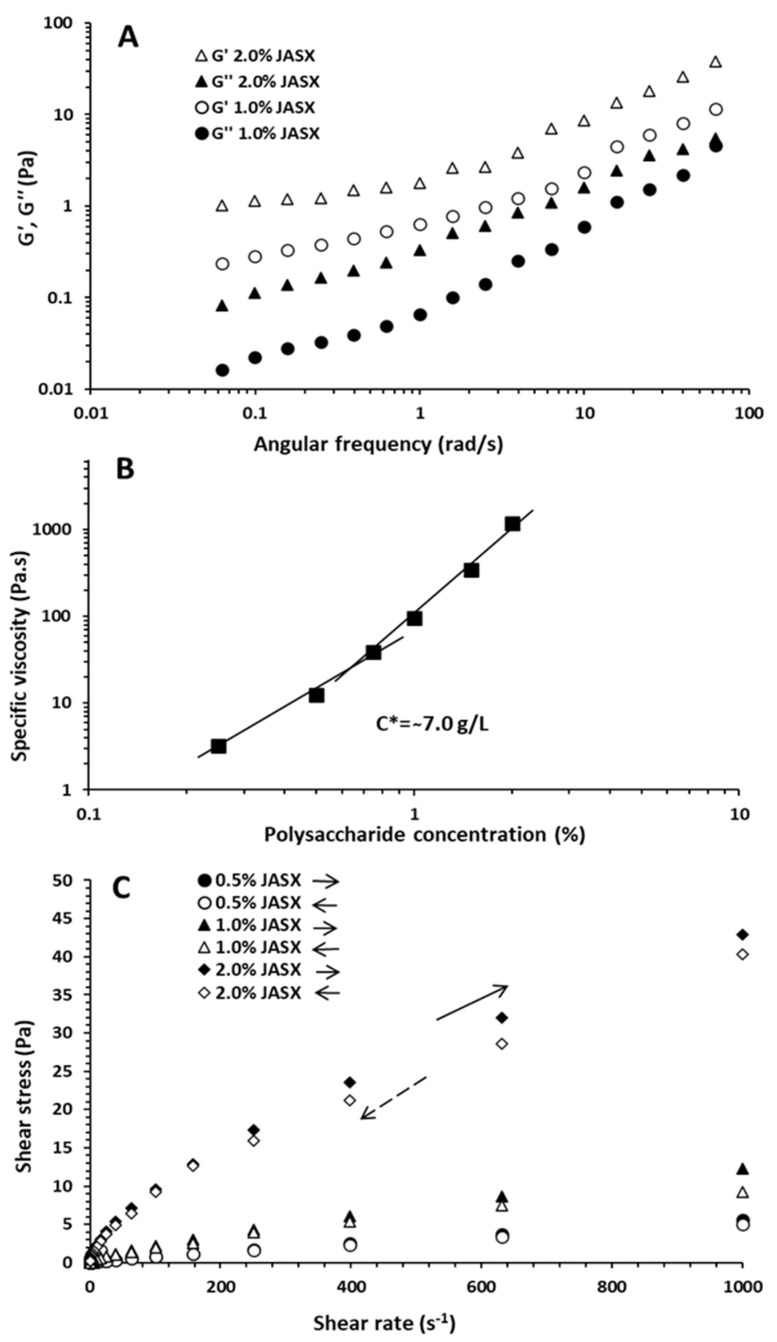
(**A**) Dynamical oscillatory properties of aqueous JASX solutions of 1.0 and 2.0% (*w*/*v*) at 25 °C. (**B**) Critical overlap (C*) concentration obtained from the log–log plot of the specific viscosity (*η_sp_*) versus the concentration of JASX in water at 25 °C. (**C**) Shear time dependence of JASX (0.5–2.0%, *w*/*v*) flow curves at 25 °C (the tracking of JASX viscosity with increasing shear rate (→, forward) and decreasing shear rate (←, return).

The dynamic oscillatory analyses were investigated on JASX solutions (1.0–2.0%, *w*/*v*) in Milli-Q water at 25 °C, and the frequency dependence of elastic modulus *G*′ (or storage modulus) and viscous modulus *G*″ (or loss modulus) are described in Figure 6A. Results showed that *G*′ and *G*″ values increased with rising polymer concentrations and angular frequencies (*ω*) (from 0.063 to 62.83 rad.s^−1^). JASX solutions exhibited typical gel-like behavior throughout the entire frequency range, with significant deformation when the *G*′ values were greater than the *G*″ values. [4,14,31]. The difference between *G*′ and *G*″ (gap) for JSAX (1.0–2.0%, *w*/*v*) increased with increasing *ω* from 0.063 to 62.83 rad.s^−1^, indicating that JASX has high viscoelasticity with a higher elastic contribution to the gel structure [4,32].

The damping factor, also known as the dynamic mechanical loss tangent (tan *δ* = *G*″/*G*′), is a characteristic parameter used to evaluate viscoelastic behavior. The tan *δ* values were inferior to 1 and confirmed the elastic behavior for JASX aqueous samples. The JASX energy was dissipated by an elastic flow, and the gel conformational equilibrium increased by favoring elastic (weak gel) behavior [32]. 

The Ostwald-de Waele model was used to describe the dependence of *G*′ and *G*″ moduli. The *n*′ values were used to determine the nature/type and strength of the gel; high *n*′ values *n*′ > 0 (*n*′ ~ 1) indicate viscous gel (physical gel), whereas low *n*′ values (zero) indicate covalent gel [29,32]. In addition, the values of *n*′ and *n*″ and *k*′ and *k*″ ones present an indicator of the nature of the polymer behavior, for *n*″ > *n*′ and *k*′ > *k*″, the system behaves as a gel, while for *n*′ > *n*″ and *k*″ > *k*′, the polysaccharide samples present a viscous-like fluid. Results showed that the *n*″ magnitudes were larger than *n*′ ones, meaning that *G*″ increased with higher rates than *G*′ (Supplementary Table S1). The *k*′ values were higher than those of *k*″, confirming the weak gel behavior of JASX. Regarding the values of *R*′^2^ and *R*″^2^, the Ostwald-de Waele model could be used to describe the viscoelastic behavior of JASX in Milli-Q water.

#### 2.2.3. Critical Overlap Concentration (C*) of JASX

The C* named critical overlap concentration denotes the boundary between dilute (non-entangled system) and semi-dilute (entangled network) media. It was calculated from the log–log plot of the *η_sp_* (specific viscosity) vs. JASX concentrations (0.5–2.0%, *w*/*v*) in Milli-Q water (Figure 6B). As illustrated in Figure 6B, the *η_sp_* increased with rising JASX concentrations and the slope break between the two linear segments allowed for evaluation of the experimental value of C* [29]. The C* value of JASX at 25 °C was estimated at 7.0 g.L^−1^ (0.7%, *w*/*v*), and the linear segment slopes below and above the C* were, respectively, 4.93 and 2.68. The theoretical C* of JASX is an inverse function of the [*η*] (mL.g^−1^) and can be calculated using the following equation: $C^{*}=k_{s}/\left[\eta \right]$, where *k_s_* is a specific constant for each type of polysaccharide [4]. For JASX in water, the *k_s_* value was calculated at around 0.714 (with C* = 7.0 g.L^−1^), which was consistent with the literature data when *k_s_* = 0.5–4 for coil polysaccharides in water [33].

#### 2.2.4. Thixotropic Properties of JASX

Solutions of several hydrocolloids are well known for their time-dependent properties, meaning that the *η* varies with shearing time [3]. Contrary to rheopexy, thixotropy is an ordinary property for non-Newtonian solutions where the *η* decreases with shearing time action [34]. According to Razmkhah et al. [30], the hysteresis loop method and the estimation of the gap values between the up and down curves were used to characterize the viscosity–time relationship of JASX (0.5–2.0%, *w*/*v*) in Milli-Q water at 25 °C. From Figure 6C, the hysteresis loops of JASX solutions presented thixotropic characteristics since the up and down curves were different [30]. The thixotropic properties tended to rise with the rise in JASX concentrations (0.5 to 2.0%, *w*/*v*), consequently suggesting that the time dependence and the damage of polymer macromolecules were stronger [3,34]. Authors such as Ma et al. [3] and Hentati et al. [14,29] demonstrated that the decrease in *η* with shearing time was primarily caused by the pseudoplastic behavior, the alignments disturbances of the polymer chain alignment disturbances, and disentanglement–entanglement processes.

### 2.3. Antioxidant Activities of JASX

#### 2.3.1. DPPH Radical-Scavenging Activity 

**Figure 7:**
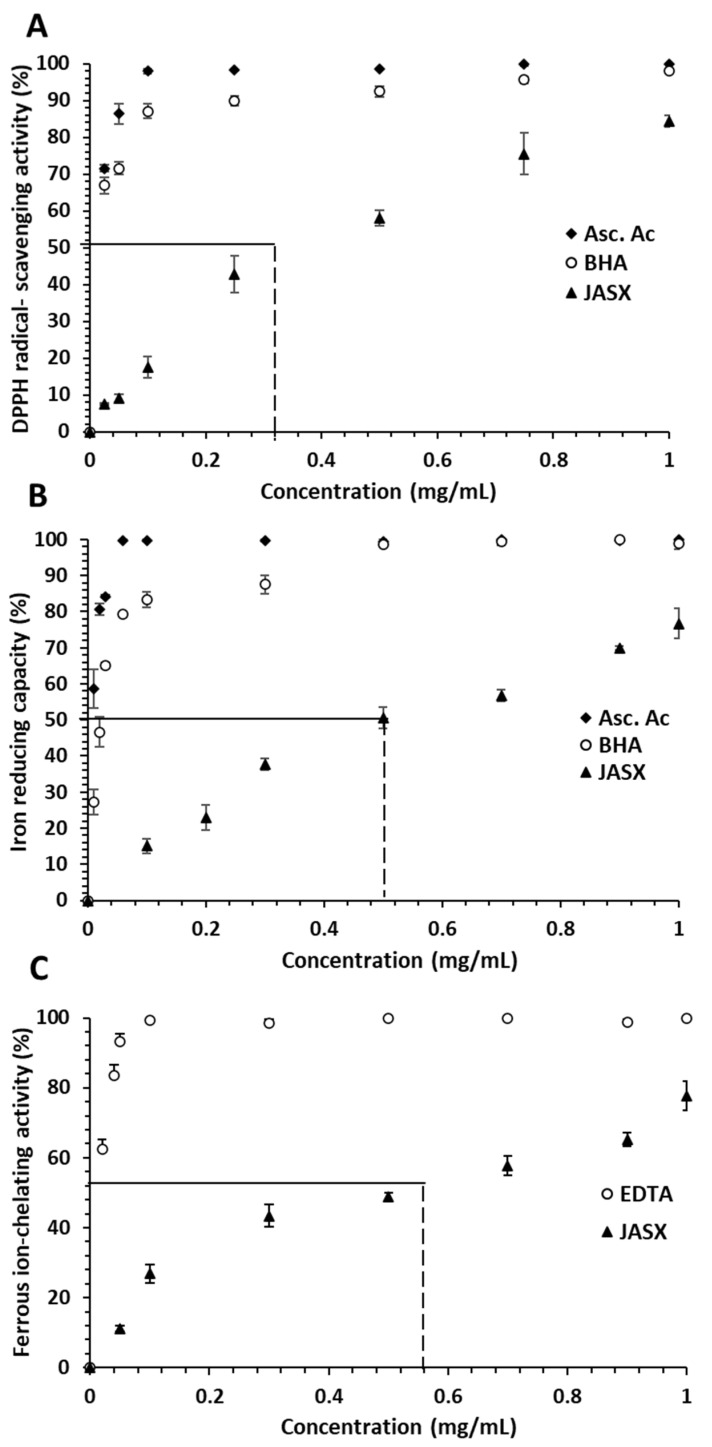
(**A**) DPPH radical-scavenging activity, (**B**) iron reducing capacity and (**C**) ferrous ion-chelating activity of JASX at different concentrations. The values are means ± SD (*n* = 3).

The DPPH radical-scavenging activity of JASX concentrations (0–1.0 mg.mL^−1^) was evaluated. Ascorbic acid and butylated hydroxyanisole (BHA) were used as positive control. Results in Figure 7A showed increasing DPPH antioxidant activity with increasing polymer concentration. 

The antiradical effect of JASX on the DPPH radical was found pronounced and concentration dependent, i.e., the 0.025 and 0.5 mg.mL^−1^ concentrations showed 7.52–58.03% of inhibition, respectively. JASX exhibited IC_50_ values near to 320 μg.mL^−1^, which were lower than those found for BHA (10 μg.mL^−1^) and ascorbic acid (7.0 μg.mL^−1^). The highest scavenging power of 84.41% was obtained at 1.0 mg.mL^−1^ of JASX. BHA and ascorbic acid showed antioxidant activities of 99.60% and 100%, respectively, at highest concentration. 

Lajili et al. [6] demonstrated that sulfated galactan extracted from the Pheophyceae *Laurencia obtusa* exhibited significant anti-DPPH ability (IC_50_ = 24 ± 5 μg.mL^−1^) close to that of ascorbic acid (17 ± 3 μg.mL^−1^) and quercetine (18 ± 2 μg.mL^−1^). According to Yang et al. [31], the anti-DPPH capacity of sulfated polysaccharide fractions (F1 and F2) obtained from the red algae *Corallina officinalis* were 15.2% at 1.2 mg.mL^−1^. At 0.6 mg.mL^−1^, JASX showed important anti-DPPH power (~25.0%) but remained inferior to the one measured for polysaccharide extracted from *G. carticata* (73%) at the same concentration [35]. However, the JASX antiradical activity (84.41% at 1.0 mg.mL^−1^) was higher than those described for *Undaria pinnitafida* (30–35%) polysaccharide fractions (S1 and S2) at 2.2 mg.mL^−1^ [36]. From the literature, DPPH radical-scavenging activity of red algal polysaccharides is highly related to their uronic sugar and 3,6-α-l-AnGal*p* content. Aside from carboxylic groups, sulfate and methyl groups, double bonds, and their conjugation to hydroxyl groups (-OH) and ketonic groups (C=O) (as in the case of ascorbic acid) also play a substantial role in their antioxidant abilities [37]. According to Abad et al. [37], the presence of two consecutive active (-OH) groups in the polymer structure may also play a role in the scavenging capacity of sulfated polysaccharides (particularly carrageenan) via the typical H-abstraction reaction with free radicals. The low molar masses of polysaccharides influenced the antioxidant power [20], which can be correlated to their reduced sugar content, the availability of functional groups and their hydrogen-donating capacity.

#### 2.3.2. Ferric-Reducing Power

The ferric ion-reducing ability assay measures the electron donating power of an antioxidant. Because of the presence of reducing agents (polysaccharides), the Fe^3+^/ferricyanide complex (ferric iron form) is reduced to the ferrous iron form (Fe^2+^). The A_700_ nm of the resulting blue-green solution is proportional to the amount of Fe^2+^ present. As a result, increased absorbance indicates greater ferric ion-reducing power. The reducing power of JASX, BHA and ascorbic acid increased with increasing concentrations (from 0 to 1.0 mg.mL^−1^) to obtain respectively reducing activities of 76.69, 98.64 and 100% at 1.0 mg.mL^−1^ (Figure 7B). Lajili et al. [6] indicated that sulfated polysaccharide from the Pheophyceae *L. obtusa* showed IC_50_ of 92 ± 2 μg.mL^−1^, which was slightly lower than those of ascorbic acid (62 ± 8 μg.mL^−1^) and quercetine (83 ± 4 μg.mL^−1^). According to Qu et al. [38] and Hentati et al. [20], the FRAP capacity of polysaccharides was related to their sulfation rate, molecular mass, content of hydroxyl and carboxylic groups of uronic acid residues.

#### 2.3.3. Ferrous Ion-Chelating Activity

Figure 7C depicts the ferrous ion-chelating activity of *J. adhaerens* sulfated xylogalactan. Through Fenton-type reactions, ferric iron (Fe^3+^) can be reduced to active Fe^2+^ and oxidized again, producing hydroxyl radicals [39]. The results showed that the JASX presented important concentration-dependent chelating capacity at 1.0 mg.mL^−1^ (78.52%), while EDTA ferrous chelating ability was 99.50% at the same concentration. The ferrous ion-chelating power enregistrated for JASX was higher than that reported by Alves et al. [40] using sulfated polysaccharides from *Hypnea musciformis*, which had 8.0% ferrous ion-chelating ability at 5.0 mg.mL^−1^. The result of 65.13% (at 0.9 mg.mL^−1^) was higher than the result of 62.46% (at 2.0 mg.mL^−1^) found by Alencar et al. [41] for sulfated polysaccharides produced from *Gracilaria caudata*. This ion-chelating capacity may be explained by the nucleophilic nature of the free electrons of hydroxyl and sulfate groups present in the polysaccharide chemical structure [20].

## 3. Material and Methods

### 3.1. Marine Seaweed Collection

Rhodophyceae *Jania adhaerens* was harvested in August 2018 from Tabarka (36°57′36.7″ N 8°45′30.3″ E, northern Tunisia). Macroalgae were washed with sea water (3 times), then with distilled water (3 times), and dried at 55 ± 1 °C for 11 days using a drying oven. The dried seaweeds were ground into a fine powder using a mechanical blender (Moulinex, France) and sieved with a mesh size of 0.3 mm. All chemicals were analytical grade and purchased from Sigma-Aldrich (St. Louis, MO, USA).

### 3.2. Extraction and Purification of Jania adhaerens Sulfated Xylogalactan (JASX)

For depigmentation, 100 g of algal powder was treated sequentially with acetone (99.5%) and ethanol (96%) for 24 h under stirring (400 rpm) at 25 °C. The depigmented biomass was then dried for 24 h at 50 ± 1 °C. The polysaccharide extraction was carried out according to the protocol proposed by Fenorodosoa et al. [1] with some modifications.

The depigmented powder (50 g.L^−1^) was dissolved in ultrapure water at 90 °C for 5 h under reflux and stirring (500 rpm). The mixture was successively treated using a fine mesh strainer, filtered through glass filters of porosity 2 (40–100 µm) and then 3 (16–40 µm). The permeate was centrifuged (12,000× *g*, 30 min, 20 °C), and the supernatant was collected before being precipitated with three volumes of −20 °C cold ethanol (96%) over 12 h at 4 °C with gentle stirring (250 rpm). The cold ethanolic precipitation and washing steps were repeated five times (using the same method) to remove salts (with conductimetry control). Samples were collected after centrifugation (8000× *g*) for 15 min at 4 °C and the final pellet was dissolved in 5-fold of milli-Q water and then freeze-dried. Finally, it was finely crushed and called *Jania adhaerens* sulfated xylogalactan (JASX).

### 3.3. Global Biochemical Composition of JASX

Carbohydrates neutral and uronic contents were evaluated according to Dubois et al. [42], Monsigny et al. [43] and Blumenkrantz and Asboe-Hansen [44]. The sulfation content was evaluated by the turbidimetric method BaCl_2_/gelatin [45]. The 3,6-α-d-AnGal*p* concentration was estimated using the method of Yaphe and Arsenault [46] (d-Fru as standard). The content of pyruvic acetal was quantified by the method of Sloneker et al. [47]. Water soluble proteins were estimated using Coomassie Brilliant Blue G-250 method [48]. Total phenolic compounds were quantified by the method of Folin–Ciocalteu using gallic acid as standard [49]. The conductivity was converted into NaCl concentration by assuming that 2 mS.cm^−1^ was equivalent to 1.0 g.L^−1^ of NaCl. Every measurement was performed three times (*n* = 3).

### 3.4. Solvolytic Desulfation of Polysaccharide

JASX aqueous solution (20 g.L^−1^) was desulfated according to the protocol proposed by Hentati et al. [14]. Briefly, sample was treated with Dowex cations exchange resin (3 h), neutralized with pyridine (pH 6.5–7) and then concentrated by rotary evaporator. The concentrated solution (15–20 mL) was mixed with acetone (99.8%, three times), concentrated again and then freeze-dried. Pyridinium salts were desulfated using DMSO/MeOH anhydrous mixture for 30 min, (500 rpm) and then boiled at reflux (3.5 h, 100 °C). The solution was neutralized (1 M NaOH, pH 9–9.5), dialyzed (against ultrapure water) and freeze-dried. The desulfated fraction of JASX was named JADX.

### 3.5. Structural Features of JASX

#### 3.5.1. ATR-FTIR Analysis

Attenuated total reflection (ATR) Fourier-Transform Infrared (FT-IR) measurements of JASX and JADX fractions were recorded on a VERTEX 70 FT-IR instrument (Ettlingen, Germany). Samples were analyzed on ATR A225 diamante (Bruker VERTEX 70, Ettlingen, Germany). The IR spectra (fifty scans) were obtained at room temperature (referenced against air) in the wavenumber range of 400–4000 cm^−1^ (resolution of 4 cm^−1^). Spectra were analyzed with OPUS 7.2 software (Bruker, Ettlingen, Germany).

#### 3.5.2. Determination of Monosaccharide Composition by GC/MS

First, 15 mg of JADX was dissolved in 1.5 mL of trifluoroacetic acid (TFA, 2M) at 120 °C for 90 min to release the monosaccharides. Polysaccharides were stirred time to time during the hydrolysis. The obtained preparation was then evaporated under nitrogen stream at 60 °C for 3 h. The monosaccharide derivatization was carried out for 2 h at 30 °C using BSTFA: TMCS (99:1) method as described by Pierre et al. [50,51]. After evaporation of the mixture (nitrogen flow), the resulting trimethylsilyl-*O*-glycosides were dissolved at 10 g.L^−1^ in dichloromethane. Following the same experimental conditions, standard monosaccharides (e.g., d-Xyl, l-Ara, d-Gal, l-Fuc, d-Man, d-Glc, l-Rha, d-Rib, d-GlcA, d-GalA, d-GlcN, d-GalN) were prepared. The samples were analyzed by gas chromatography–mass spectrometry (GC/MS) coupled to electronic impact (EI) using an Agilent 6890 Series GC System coupled to an Agilent 5973 Network Mass Selective Detector (Agilent Technologies, Les Ulis, France). Then, 1 µL of derivatives (sample/standard) was injected on an OPTIMA-1 MS (30 m, 0.32 mm, 0.25 μm) column with a helium flow rate of 2.3 mL.min^−1^. The different stages of temperature increase were carried out according to the method described by Hentati et al. [14]. The ionization step was carried out by EI (70 eV) with the trap temperature set at 150 °C and the target ion set at 40–800 *m*/*z*. The injector temperature was set to 250 °C, the split ratio to 50:1, and the helium pressure to 8.8 psi.

#### 3.5.3. SEC-MALLS Analysis of JASX

HPSEC (high-pressure size exclusion chromatography) was used to estimate macromolecular magnitudes (the mass average molar mass (M_w_), number average molar mass (M_n_), polydispersity index (Đ) = M_w_/M_n_, hydrodynamic radius (R_h_) and intrinsic viscosity ([*η*])) of JASX in the diluted regime. HPSEC was equipped with three detectors in line: (i) a multi-angle laser light scattering (MALLS) filled with a He–Ne laser (λ = 690 nm) and a K5 cell (50 μL) (HELEOSII Wyatt Technology Corp., Goleta, CA, USA), (ii) a viscosimeter (Viscostar II, Wyatt Technology Corp., Santa Barbara, CA, USA), and (iii) a differential refractive index (DRI) (RID10 A Shimadzu, Kyoto, Japan). The solvent was filtered through 0.1 μm filter unit (Millipore), degassed (DGU-20A3, Shimadzu, Kyoto, Japan) and filtered through an upstream 0.45 m filter column. The SEC line was composed of an OHPAK SB-G guard column and two OHPAK SB 806 and 804 HQ columns (Shodex Showa Denko K.K, Tokyo, Japan) eluted in series with LiNO_3_ solution (0.1 M) at a flow rate of 0.7 mL.min^−1^. JASX was solubilized for 24 h at a concentration of 2 g.L^−1^ in 0.1 M LiNO_3_ solution, filtered (0.45 μm) and then injected through a 500 μL full loop (SIL-20A, Shimadzu, Kyoto, Japan). The concentrations of the samples were calculated using a differential refractive index dn/dc of 0.15 mL.g^−1^. Astra 6.1 software was used to analyze the data.

#### 3.5.4. NMR Spectroscopy Analysis

JASX (sulfated fraction) and JADX (desulfated fraction) were prepared in D_2_O (99.9% D) at 60 g.L^−1^ and then freeze-dried. Before analysis, polysaccharides were solubilized at 40 g.L^−1^ in D_2_O. NMR spectra were collected at 60 °C using a Bruker Avance 600 spectrometer (Bruker BioSpin MRI GmbH, Ettlingen, Germany) operating at 600 MHz and equipped with a Broad Band Fluorine Observation (BBFO) probe. NMR experiments were performed with a spectral width of 3000 Hz using the following acquisition parameters: (i) for ^1^H analyses, acquisition mode = 2 s, recovery = 5 s (for a complete return after a 90° pulse), number of scans = 64 and pulse 90° = 8 µsec and (ii) for ^13^C experiments, number of scans = 16,384, acquisition mode = 0.34 s, recovery = 2 s, pulse = 7 µs, accumulation for 11 h.

### 3.6. Rheological Investigations

#### 3.6.1. Rheological Measurements

JASX solutions at concentrations ranging from 0.25 to 2.0% (*w*/*v*) were made by soaking dried samples in Milli-Q water or 0.5 M NaCl solutions for 4 h (at room temperature) and gently stirring until full dissolution. After that, samples were kept at 4 °C for 48 h to achieve a fully water-swelling polymer (biopolymer hydration) and to remove bubbles.

Rheological measurements on JASX solutions were carried out with a rheometer AR-2000 (TA Instrument, Great Britain, Ltd. New Castle, DE, USA ) fitted with a 40 mm cone-plate geometry (54 microns gap) and a Peltier heating system for precise control. To prevent solvent evaporation during rheological analysis, samples were covered with a thin layer of hexadecane (oil film) after structure recovery and temperature equilibration (15 min). The TA Instrument Rheology Advantage software was used to collect and analyze the data (V5.7.0).

#### 3.6.2. Steady-Shear Flow Measurements

The cone-plate geometry was used to investigate the steady-shear flow properties of JASX solutions (0.25–2.0%, *w*/*v*) at 25 °C over the shear rate ($\dot{\gamma }$) range from 0.001 to 1000 s^−1^. The shear stress (*τ*) and dynamic viscosity (apparent viscosity, *η*) were measured as a function of $\dot{\gamma }$ in different conditions (in Milli-Q water and in 0.5 M NaCl) by applying the following model (Equation (1)):

(1) $$\eta =\tau /\dot{\gamma }$$

To fit the experimental rheological data of JASX, the Ostwald-de Waele model (power-law model) (Equation (2)) was used.

(2) $$\tau =k.{\dot{\gamma }}^{n}$$

where *τ*: the shear stress (Pa); *k*: the consistency index (Pa.s^n^); $\dot{\gamma }:\;$the shear rate (s^−1^) and *n*: the flow behavior index (dimensionless), which takes the values >1, 1 and <1 for plastic, Newtonian and pseudoplastic fluid behaviors, respectively.

The temperature-dependent viscosity of JASX (1.0–2.0% (*w*/*v*)) was measured at shear rates ranging from 1.0 to 1000 s^−1^ in the temperature range of 20 to 45 °C using the Arrhenius–Frenkel–Eyring model (Equation (3)):

(3) $$\eta =A\exp\left(\frac{E_{a}}{RT}\right)$$

where *η*: the apparent viscosity (Pa.s); *A*: the proportionality constant (Pa.s); *T*: the absolute thermo-dynamical temperature (K); *R*: the universal gas constant (kJ.mol^−1^·K^−1^) and *E_a_*: the flow activation energy (kJ.mol^−1^).

The Williamson model (Equation (4)) was used to calculate the critical overlap concentration (C*) of JASX aqueous solutions (0.25–2.0%, *w*/*v*) at 25 °C, which represents the limit between dilute (non-entangled system) and semi-dilute (entangled network) regimes.

C* was calculated using a log–log plot of specific viscosity (*η_sp_*) versus polysaccharide concentration.

(4) $$\eta =\frac{\eta_{s}}{(1+\left(\lambda .\dot{\gamma }{)}^{1-n}\right)}$$

where *η*: the apparent viscosity (Pa.s); *η_s_*: the zero-shear viscosity (Pa.s); *λ*: the transition time (or time constant, s); $\dot{\gamma }$: the shear rate (s^−1^) and *n*: the flow behavior index (dimensionless).

#### 3.6.3. Dynamic Viscoelastic Properties

The oscillatory (dynamic) frequency sweeps for JASX (1.0–2.0%, *w*/*v*) were evaluated in a constant strain of 20% (or in linear viscoelastic range) at 25 °C using the cone-plate geometry over angular frequency (*ω*) ranging from 0.063 to 62.83 rad.s^−1^ (0.01 to 10 Hz). The storage or elastic modulus *G*′, the loss or viscous modulus *G*″ and the loss tangent or damping factor (tan *δ* = *G*″/*G*′) as a function of *ω* were continuously determined during the rheological analysis. The power-law model (Equations (5) and (6)) was used to describe the frequency dependence of the moduli *G*′ and *G*″:

(5) $$G\prime =k\prime \;{\left(\omega \right)}^{n^{\prime }}$$

(6) $$G\prime \prime =k\prime \prime \;{\left(\omega \right)}^{n^{\prime \prime }}$$

where *k*′ and *k*″: the specific constants; *n*′ and *n*″: the frequency exponents and *ω*: the angular frequency (rad.s^−1^ or Hz).

### 3.7. Antioxidant Activity 

#### 3.7.1. DPPH Radical-Scavenging Activity

The method of Kirby and Schmidt [52] was used to evaluate the DPPH (2,2-diphenyl-1-picrylhydrazyl) radical-scavenging ability of JASX. Briefly, 500 μL of samples (0–1.0 mg.mL^−1^) was mixed with ethanol (99%, 375 μL) and a DPPH diluted solution in ethanol (125 μL, 0.02% (*w*/*v*)). The mixture was homogenized, and then, the A_517_ of samples were measured after incubation at 25 °C during 30 min in the dark. BHA and ascorbic acid were used as positive standards, and then, the DPPH scavenging activity was calculated using Equation (7).

(7) $$\mathrm{DPPH}\;\mathrm{scavenging}\;\mathrm{power}\;\left(\%\right)=\left[\frac{A_{control}-\left(A_{sample}-A_{blank}\right)\;}{A_{control}}\right]\times 100$$

where *A_control_*: the absorbance of the control reaction (absence of polysaccharide); *A_blank_*: the absorbance of JASX samples (except the DPPH solution); *A_sample_*: the absorbance of JASX in DPPH solution.

#### 3.7.2. Ferric-Reducing Power

The Ferric-reducing activity of JASX was determined according to the method of Yildirim et al. [53]. First, 0.5 mL of polysaccharide solutions (0–1.0 mg.mL^−1^) were mixed with 1.25 mL of phosphate buffer (0.2 M, pH = 6.6) and 1.25 mL of potassium ferricyanide (1% (*m*/*v*)). Then, the mixture was incubated for 30 min at 50 °C, treated with 1.25 mL of trichloroacetic acid (10% *w*/*v*) and centrifuged 3000 g during 10 min. Next, 1.25 mL of the supernatant was thoroughly mixed with 1.25 mL of ultrapure water and 0.25 mL of a 0.1% (*w*/*v*) ferric chloride solution. After incubation (10 min, 25 °C), the A_700_ was measured. Butylated hydroxyanisole (BHA) and ascorbic acid were used as standards, and then, the FRAP capacities were calculated using Equation (8). All the experiments were performed in triplicate.

(8) $$\mathrm{Ferric}\;\mathrm{reducing}\;\mathrm{ability}\;\left(\%\right)=100-\left(\left(\frac{A_{0}-A_{sample}\;}{A_{0}}\right)\times 100\right)$$

where *A*_0_: the absorbance of a 66 μM Prussian blue solution measured in the same reaction medium free of reducing component (*A*_0_ = 0.8) and *A_sample_*: the absorbance of JASX samples.

#### 3.7.3. Ferrous Ion-Chelating Power

The chelating capacities of ferrous ions by JASX were determined following the method of Carter [54]. FeCl_2_ solution (100 μL, 2mM) was added to 200 μL of different polysaccharide concentrations (0–1.0 mg.mL^−1^). After incubation (5 min, 25 °C), the reactions were triggered by adding 400 μL of ferrozine solution (5 mM), and the preparations were vigorously shaken and then incubated (10 min, 25 °C). The inhibition ratio of the ferrozine–Fe^2+^ complex formation was determined following Equation (9) after measuring the A_562_.

(9) $$\mathrm{Ferrous}\;\mathrm{ion}\;\mathrm{chelating}\;\mathrm{activity}\;\left(\%\right)=\left[\frac{A_{control}-\left(A_{sample}-A_{blank}\right)\;}{A_{control}}\right]\times 100$$

where the absorbance of the control (without sample), the absorbance of the blank (without Ferrozine) and finally the absorbance of the extract respectively represented *A_control_*, *A_blank_* and *A_sample_*. The positive control test was performed by EDTA (ethylenediaminetetraacetic acid).

## 4. Conclusions

The main goals of this paper were to determine the structural features and the rheological properties and to evaluate the antioxidant activities of a sulfated xylogalactan produced from the red seaweed *J. adhaerens*. The structural analyses revealed that JASX (M_w_ = 6.0 × 10^5^ Da) was mainly composed of an agar-like sulfated xylogalactan with a repeating backbone of (→3)-β-d-Gal*p*-(1,4)-3,6-α-l-AnGal*p*-(1→)_n_ and (→3)-β-d-Gal*p*-(1,4)-α-l-Gal*p*-(1→)_n_ substituted on *O*-2 and *O*-3 of the α-(1,4)-l-Gal*p* units by methoxy and/or sulfate groups but also on *O*-6 of the β-(1,3)-d-Gal*p* mainly by β-xylosyl side chains and less by methoxy and/or sulfate groups. The rheological investigations showed that JASX solutions exhibited a shear-thinning behavior influenced by temperature and adding salts. JASX presented thixotropy properties and a critical overlap concentration C* close to 7.0 g.L^−1^. The dynamical viscoelastic properties showed a gel-like viscoelastic behavior (*G*′ > *G*″) with a great viscoelastic character. The physicochemical and antioxidant properties of JASX are at least as good as other polysaccharides from red marine seaweeds currently used for their functional properties as hydrocolloids.

## Figures and Tables

**Table 1:** Global composition of JASX polysaccharide extracted from *Jania adhaerens*.

Extraction Yield (%, *w*/*w*)	Total Sugar (% *w*/*w*)	Neutral Sugar (% *w*/*w*)	Uronic Acid (% *w*/*w*)	Proteins (% *w*/*w*)	Phenolic Compounds (% *w*/*w*)	Sulfate (% *w*/*w*)	Pyruvate (% *w*/*w*)	3,6-AnGal (% *w*/*w*)	NaCl (%)
5.25	73.52 ± 0.85	68.02 ± 0.76	6.55 ± 0.61	0.64 ± 0.06	0.66 ± 0.02	12.55 ± 0.61	0.35 ± 0.04	19.53 ± 1.16	1.25

**Table 2:** Monosaccharide composition and macromolecular characteristics of JASX.

Monosaccharide Composition (Molar %) ^a^				M_w_^b^ (kDa)	M_n_^c^ (kDa)	Đ ^d^	R_h_^e^ (nm)	[*η*] ^f^ (mL.g^−1^)	C* ^g^ (g.L^−1^)
Gal*p*	Xyl*p*	Glc*p*	Glc*p*A						
73.06	16.66	8.46	1.81	600	160	3.7	17.2	102	7.0

^a^ Monosaccharide composition was determined by GS/MS-EI. ^b^ M_w_: mass average molecular mass was measured by SEC-MALLS-DRI. ^c^ M_n_: number of average molecular mass. ^d^ PDI: polydispersity index (M_w_/M_n_) was estimated by SEC-MALLS-DRI. ^e^ R_h_: hydrodynamic radius was calculated by SEC Viscosity. ^f^ [*η*]: intrinsic viscosity was measured by SEC Visco-DRI. Analyses were run in triplicate, and the relative standard deviations are less than 5%. ^g^ C*: critical overlap concentration was determined using the Williamson model.

**Table 3:** Consistency and flow behavior index of JASX solutions in water and monovalent salts.

JASX (%, *w*/*v*)	NaCl (mol.L^−1^)	*n*	*k* (Pa.s^n^)	*R* ^2^
**0.25**	0.0	0.70 ± 0.014	0.018 ± 0.001	0.98
	0.5	0.75 ± 0.022	0.015 ± 0.004	0.98
**0.50**	0.0	0.66 ± 0.009	0.038 ± 0.002	0.99
	0.5	0.69 ± 0.013	0.032 ± 0.001	0.98
**0.75**	0.0	0.59 ± 0.015	0.118 ± 0.009	0.99
	0.5	0.64 ± 0.032	0.103 ± 0.005	0.97
**1.0**	0.0	0.56 ± 0.024	0.187 ± 0.012	0.99
	0.5	0.60 ± 0.019	0.162 ± 0.008	0.98
**1.5**	0.0	0.53 ± 0.005	0.417 ± 0.023	0.99
	0.5	0.57 ± 0.017	0.399 ± 0.018	0.99
**2.0**	0.0	0.52 ± 0.021	0.871 ± 0.044	0.99
	0.5	0.55 ± 0.034	0.852 ± 0.032	0.98

**Table 4:** Arrhenius–Frenkel–Eyring relationship fitting parameters (*Ea* and *R*^2^) for JASX solutions of 1.0–2.0% (*w*/*v*) at different shear rates ranging from 1 to 1000 s^−1^.

JASX (%, *w*/*v*)	Parameters	Shear Rate (s^−1^)						
		1	3.981	10	39.81	100	398.1	1000
1.0	*E_a_* (Kcal.mol^−1^)	5.50	5.10	5.03	4.72	4.63	4.20	4.02
	*R* ^2^	0.99	0.99	0.99	0.99	0.99	0.99	0.99
1.5	*E_a_* (Kcal.mol^−1^)	5.49	5.11	5.03	4.70	4.63	4.17	3.99
	*R* ^2^	0.99	0.99	0.99	1.00	0.99	0.99	0.99
2.0	*E_a_* (Kcal.mol^−1^)	5.47	5.02	4.93	4.67	4.58	4.13	3.92
	*R* ^2^	0.99	0.99	0.99	0.99	0.99	0.99	1.00

## Data Availability

The data presented in this study are available on request from the corresponding author.

## Supplementary Materials

The following supporting information can be downloaded at: https://www.mdpi.com/article/10.3390/md20090546/s1, Figure S1: FT-IR spectra of (A) JASX (sulfated fraction) and (B) JADX (desulfated fraction); Table S1: Power-law model fitting viscoelastic parameters for JASX (1.0–2.0%, *w*/*v*) solutions in water.
