# Supplementary material for: Structural Characterization and Rheological and Antioxidant Properties of Novel Polysaccharide from Calcareous Red Seaweed
Source: Mar Drugs. 2022 Aug 25;20(9):546. doi: 10.3390/md20090546 (PMC9504466; doi:10.3390/md20090546)
Supplement: Supplementary file 1 [file marinedrugs-20-00546-s001.zip › marinedrugs-1863366-supplementary.pdf]

## Supplementary Data

## Figure

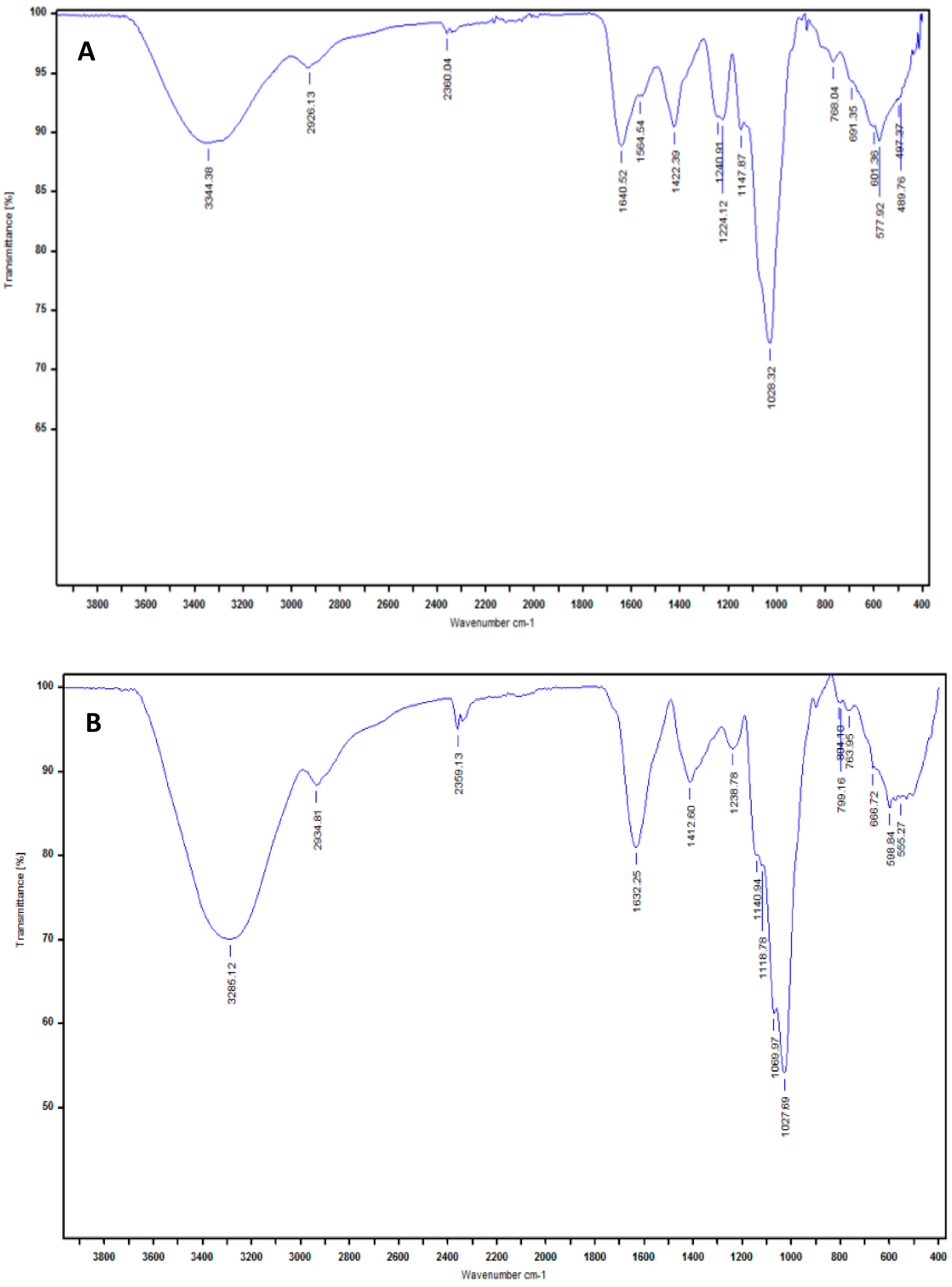


**Figure S1.** FT-IR spectra of **(A)** JASX (sulfated fraction) and **(B)** JADX (desulfated fraction).

## Table

**Table S1.** Power-law model fitting viscoelastic parameters for JASX (1.0-2.0%, *w*/*v*) solutions in water.

| **JASX (%, *w*/*v*)** | **Power-law model fitting parameters** | | | | | |
|---|---|---|---|---|---|---|
| | $n'$ | $n''$ | $k'$ | $k''$ | $R'^2$ | $R''^2$ |
| 1.0 | 0.538 ± 0.010 | 0.822 ± 0.023 | 0.729 ± 0.017 | 0.099 ± 0.001 | 0.99 | 0.97 |
| 2.0 | 0.474 ± 0.014 | 0.623 ± 0.009 | 2.703 ± 0.031 | 0.393 ± 0.021 | 0.95 | 0.99 |
